# The GD-1 Stellar Stream Perturber as a Core-collapsed Self-interacting Dark Matter Halo

**DOI:** 10.3847/2041-8213/ada02b

**Published:** 2025-01-03

**Authors:** Xingyu Zhang, Hai-Bo Yu, Daneng Yang, Ethan O. Nadler

**Affiliations:** 1 Department of Physics, Tsinghua University, Beijing 100084, People’s Republic of China; zhang-xy19@mails.tsinghua.edu.cn; 2 Department of Physics and Astronomy, University of California, Riverside, CA 92521, USA; haiboyu@ucr.edu, danengy@ucr.edu; 3 Purple Mountain Observatory, Chinese Academy of Sciences, Nanjing 210023, People's Republic of China; 4 Carnegie Observatories, 813 Santa Barbara Street, Pasadena, CA 91101, USA; enadler@carnegiescience.edu; 5 Department of Physics & Astronomy, University of Southern California, Los Angeles, CA 90007, USA; 6 Department of Astronomy & Astrophysics, University of California, San Diego, La Jolla, CA 92093, USA

## Abstract

The GD-1 stellar stream exhibits spur and gap structures that may result from a close encounter with a dense substructure. When interpreted as a dark matter subhalo, the perturber is denser than predicted in the standard cold dark matter (CDM) model. In self-interacting dark matter (SIDM), however, a halo could evolve into a phase of gravothermal collapse, resulting in a higher central density than its CDM counterpart. We conduct high-resolution controlled *N*-body simulations to show that a collapsed SIDM halo could account for the GD-1 perturber's high density. We model a progenitor halo with a mass of 3 × 10^8^* M*_⊙_, motivated by a cosmological simulation of a Milky Way analog, and evolve it in the Milky Way's tidal field. For a cross section per mass of *σ*/*m* ≈ 30–100 cm^2^ g^−1^ at ${V}_{{\mathrm{\max }}}\unicode{x0007E}10\,{\mathrm{km}}\,{{\mathrm{s}}}^{-1}$, the enclosed mass of the SIDM halo within the inner 10 pc can be increased by more than 1 order of magnitude compared to its CDM counterpart, leading to a good agreement with the properties of the GD-1 perturber. Our findings indicate that stellar streams provide a novel probe into the self-interacting nature of dark matter.

## 1. Introduction

Stellar streams form when globular clusters or dwarf galaxies are tidally stripped. There are more than 100 streams discovered in the Milky Way; see, e.g., A. Bonaca & A. M. Price-Whelan (2024), T. S. Li et al. (2022), N. Shipp et al. (2018), and references therein. Among them, the GD-1 stream is one of the longest and coldest streams (C. J. Grillmair & O. Dionatos 2006), and it has been used to constrain the Milky Way's gravitational potential (S. E. Koposov et al. 2010; J. Bovy et al. 2016; A. Bonaca & D. W. Hogg 2018; K. Malhan & R. A. Ibata 2019). The GD-1 stream has rich structural properties, such as the gaps (e.g., R. G. Carlberg & C. J. Grillmair 2013; T. J. L. de Boer et al. 2018, 2020; N. Banik et al. 2021; K. Malhan et al. 2022) and spur (A. M. Price-Whelan & A. Bonaca 2018; A. Bonaca et al. 2019, 2020), suggesting that it has been perturbed through interactions with a substructure in the Milky Way.

In particular, A. Bonaca et al. (2019) demonstrated that the perturber must be surprisingly dense to account for the spur and gap features in the GD-1 stream. Assuming a Hernquist density profile, the perturber's mass is estimated to be in the range of 10^5.5^–10^8^* M*_⊙_, with a scale radius of ≲20 pc; recent encounters within the last 1 Gyr are favored. The perturber is significantly denser than the subhalos predicted in the standard cold dark matter (CDM) model, at the ~3*σ* level. Thus, even if the perturber were a known satellite galaxy of the Milky Way, its unusually high density would remain puzzling. Furthermore, none of the known globular clusters can match the orbit of the inferred perturber (A. Bonaca et al. 2019; Y. Doke & K. Hattori 2022).

In this work, we assume that the GD-1 perturber is a dark matter subhalo and explore its formation in within the framework of self-interacting dark matter (SIDM); see S. Tulin & H.-B. Yu (2018) and S. Adhikari et al. (2022) for reviews and references therein. The gravothermal evolution of an SIDM halo occurs in two sequential phases. In the core-forming phase, dark matter self-interactions transport heat inward, resulting in a shallow density core, while in the core-collapsing phase, heat transfer reverses, leading to a higher central density than in the CDM counterpart (e.g., S. Balberg et al. 2002; J. Koda & P. R. Shapiro 2011; R. Essig et al. 2019; W.-X. Feng et al. 2021). Notably, SIDM models with large cross sections could explain the high density of the strong lensing perturber for SDSSJ0946+1006 (S. Vegetti et al. 2010; Q. E. Minor et al. 2021; E. O. Nadler et al. 2023) and the low density of the Crater II satellite galaxy (A. Borukhovetskaya et al. 2022; X. Zhang et al. 2024), both challenging CDM. It is intriguing to explore the SIDM scenario to account for the high density of the GD-1 perturber.

We will analyze progenitors of CDM subhalos from a zoom-in cosmological simulation of a Milky Way analog from D. Yang et al. (2023a) and E. O. Nadler et al. (2020b) and explicitly show that their inner densities are systematically lower than those inferred for the GD-1 perturber. We then take one of the progenitor halos, with a mass of ~10^8^* M*_⊙_, and evolve it in the tidal field of the Milky Way, including both halo and stellar components. For a self-interacting cross section in the range *σ*/*m* = 30–100 cm^2^ g^−1^ at ${V}_{{\mathrm{\max }}}\unicode{x0007E}10\,{\mathrm{km}}\,{{\mathrm{s}}}^{-1}$, the SIDM halo enters the collapse phase within 3–6 Gyr while evolving in the tidal field. By the final snapshot, its enclosed mass within the inner 10 pc is increased by more than 1 order of magnitude compared to its CDM counterpart, making it consistent with the high density of the GD-1 perturber. Additionally, we will discuss future investigations aimed at further improvement.

The rest of this Letter is organized as follows: In Section 2, we discuss the properties of CDM halos in the cosmological zoom-in simulation of a Milky Way analog. In Section 3, we introduce the setup of our *N*-body simulations. In Section 4, we present the properties of our simulated SIDM and CDM subhalos and compare them with the GD-1 perturber. In Section 5, we discuss future investigations for further improvement and conclude. In Appendix A, we present the SIDM simulation of an isolated halo for testing numerical artifacts that could lead to violation of energy conservation. In Appendix B, we show the convergence test.

## 2. CDM Halos of a Milky Way Analog

We first present progenitor halos from a cosmological zoom-in CDM-only simulation of a Milky Way analog (E. O. Nadler et al. 2020b; D. Yang et al. 2023a), with initial conditions drawn from the suite in Y.-Y. Mao et al. (2015). This simulated system includes a main halo with a mass of 1.14 ×10^12^* M*_⊙_* h*^−1^ ≈ 1.6 × 10^12^* M*_⊙_(*h* = 0.7) and a Large Magellanic Cloud analog. The simulation has a particle mass of 4 ×10^4^* M*_⊙_* h*^−1^, a Plummer-equivalent softening length of *ϵ* = 0.08 kpc *h*^−1^, and a spline length of *ℓ* = 2.8*ϵ* =0.22 kpc *h*^−1^, the characteristic length scale of the smoothing kernel used to calculate gravitational forces between particles (V. Springel et al. 2001; V. Springel 2005).

We select subhalos of the main halo with the virial mass larger than 10^8^* M*_⊙_* h*^−1^ at *z* = 0 and then identify their progenitors at infall. With the mass cut, there will be at least 2500 simulation particles for each progenitor halo so that we can accurately reconstruct its density profile. The radial resolution of the cosmological simulation *ℓ* ≈ 0.3 kpc is more than 1 order of magnitude larger than the radial scale relevant for the GD-1 perturber. To overcome this resolution limit, we fit each progenitor halo with a truncated Navarro–Frenk–White (NFW) profile (R. Errani & J. F. Navarro 2021) for the region *r* > 0.3 kpc:

\begin{eqnarray*}\rho (r)=\frac{{\rho }_{s}}{\left(r/{r}_{s}\right){\left(1+r/{r}_{s}\right)}^{2}}\times \frac{\exp (-r/{r}_{{\mathrm{cut}}})}{{(1+{r}_{s}/{r}_{{\mathrm{cut}}})}^{0.3}},\end{eqnarray*}

where *ρ*_*s*_ and *r*_*s*_ are the scale density and radius, respectively, and *r*_cut_ is the truncation radius due to tidal stripping. We determine the three parameters for each progenitor at infall, achieving excellent overall fit quality. The truncated NFW profile is then extrapolated inward to compute the total enclosed mass within *r* = 10 pc. Additionally, we confirm that many progenitor halos can be well-fitted with the standard NFW profile, while some exhibit density profiles slightly steeper than *r*^−3^ in the outer regions. For these cases, the standard NFW fit may introduce bias and overestimate the central density. However, the truncated NFW profile provides a significantly better fit.

**Figure 1.**
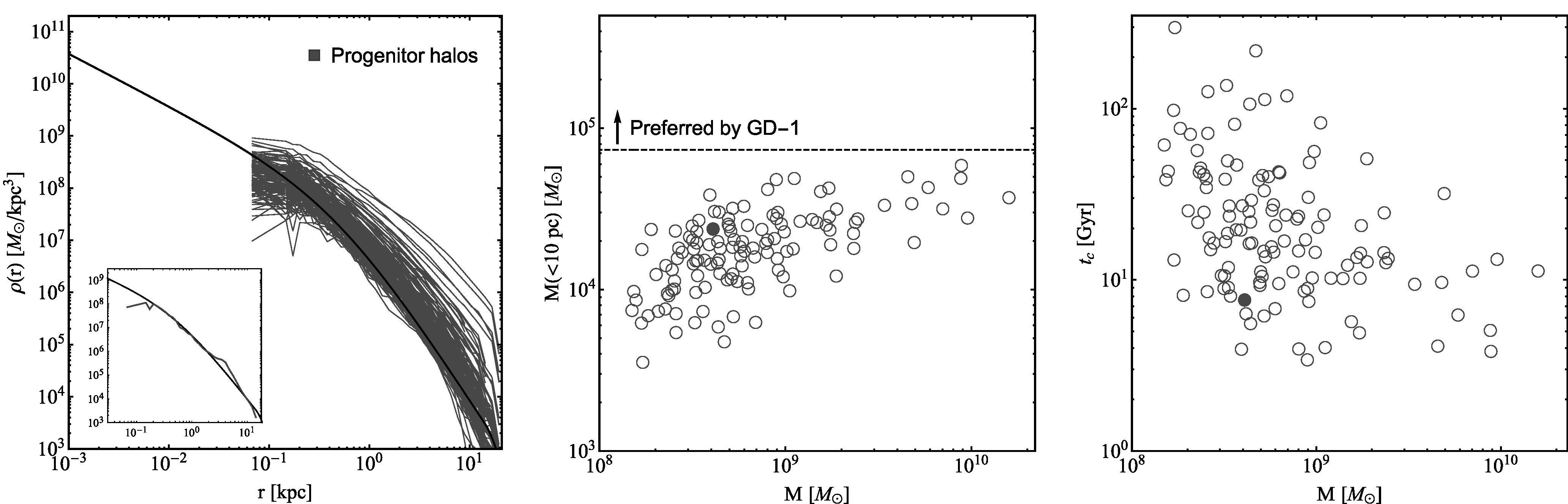
Left: density profiles for 125 CDM progenitor halos at their infall times from the zoom-in cosmological simulation of a Milky Way analog in D. Yang et al. (2023a; blue). The black line indicates an NFW density profile fitted to one of the progenitor halos (see the inset panel), which will be used as the initial condition for our controlled SIDM and CDM simulations. Middle: enclosed mass within inner 10 pc vs. virial mass for the CDM progenitor halos. The filled circle marks the halo used for the initial condition. The horizontal dashed gray line indicates the inner mass within 10 pc for a reference Hernquist profile with the scale radius *r*_H_ = 15 pc and the total mass *M* = 4.6 × 10^5^* M*_⊙_, representing one of the least dense perturber models for the GD-1 stellar stream in A. Bonaca et al. (2019). Right: estimated SIDM collapse timescale vs. virial mass for the progenitor halos, assuming *σ*/*m* = 50 cm^2^ g^−1^. The filled circle marks the halo used for the initial condition in our controlled simulations.

Figure 1 (left) shows the density profiles for the 125 progenitor halos at infall (blue). We also present the fit to one of the progenitors (black), which will be used as the initial condition for our SIDM simulations; see the detailed comparison in the inset panel. For this halo, the standard NFW profile provides a good fit. The simulated density profile is flattened for *r* ≲ 0.3 kpc due to the resolution limit. However, we expect that the NFW profile provides a good approximation for extrapolating the density inward before the halo undergoes significant tidal stripping.

Figure 1 (middle) shows the enclosed mass within 10 pc versus virial mass of the progenitor halos at infall. For comparison, we include a reference case from the viable parameter region of the GD-1 perturber in A. Bonaca et al. 2019 (their Figure 6): a Hernquist scale radius of *r*_H_ = 15 pc and a total mass of *M* = 4.6 × 10^5^* M*_⊙_, which approximately corresponds to a substructure with the minimum density required to explain the spur and gap features of the GD-1 stream. For this reference case, the enclosed mass within 10 pc is ≈7.4 × 10^4^* M*_⊙_, as denoted by the horizontal line in the middle panel. We see that none of the CDM progenitor halos are sufficiently dense to be the perturber, and this conclusion holds when comparing the enclosed mass within *r* = 15 pc. The inner density of these CDM halos would further decrease as they evolve within the Milky Way's tidal field. The progenitor CDM halos shown in Figure 1 correspond to subhalos with masses >10^8^* M*_⊙_* h*^−1^ at *z* = 0. We plan to relax this mass threshold and examine halos with lower masses. Based on the resolution limit in the cosmological CDM simulation (D. Yang et al. 2023a), we expect to reconstruct the density profiles of progenitors for subhalos with masses a few times 10^7^* M*_⊙_* h*^−1^ using the truncated NFW profile in Equation (1). A more detailed investigation will be deferred to future work.

For the CDM progenitor halos, we estimate the timescale of gravothermal collapse in SIDM (J. Pollack et al. 2015; R. Essig et al. 2019):

\begin{eqnarray*}{t}_{c}=\frac{150}{C}\frac{1}{{r}_{s}{\rho }_{s}\left({\sigma }_{{\mathrm{eff}}}/m\right)}\frac{1}{\sqrt{4\pi G{\rho }_{s}}},\end{eqnarray*}

where *C* = 0.75 is a numerical factor and *σ*_eff_ is the effective cross section (D. Yang & H.-B. Yu 2022). For simplicity, we assume a constant cross section in this work. Figure 1 (right) shows the collapse time versus virial mass for the progenitors, where we have taken *σ*/*m* = 50 cm^2^ g^−1^. About one-third of the halos are expected to collapse within 10 Gyr. Since tidal stripping could speed up the onset of the collapse (F. Kahlhoefer et al. 2019; H. Nishikawa et al. 2020; O. Sameie et al. 2020; D. Yang & H.-B. Yu 2021; Z. C. Zeng et al. 2022), we expect that more halos would be in the collapse phase after they evolve in the tidal field, and their overall mass would be reduced as well.

## 3. Simulation Setup

In this section, we introduce our simulation setup, including the initial halo density profile, the SIDM cross section, orbital parameters, and the gravitational potential model of the Milky Way.

### 3.1. The Initial Halo Density Profile and Cross Section

We choose the CDM progenitor with the earliest infall time among the five halos that have *t*_*c*_ < 10 Gyr, and its density profile is shown in Figure 1 (left, black). For this halo, the fitted NFW parameters are *ρ*_*s*_ = 7.5 × 10^7^* M*_⊙_ kpc^−3^ and *r*_*s*_ = 0.50 kpc. The maximum circular velocity and the associated radius are ${V}_{{\mathrm{\max }}}=14.8\,{\mathrm{km}}\,{{\mathrm{s}}}^{-1}$ and ${r}_{{\mathrm{\max }}}=1.1\,{\mathrm{kpc}}$, respectively. We use the public code SpherIC (S. Garrison-Kimmel et al. 2013) to generate the initial condition, and the total halo mass is *M* = 3.25 × 10^8^* M*_⊙_. The simulation has a particle mass of 32.5* M*_⊙_, a total number of 10^7^ particles, and a softening length of *ϵ* = 2 pc. We use the public *N*-body code GADGET-2 (V. Springel et al. 2001, 2005) implemented with an SIDM module from D. Yang et al. (2020), which follows the algorithm in A. Robertson et al. (2017) with small modifications.

As indicated in Figure 1 (right), the halo would collapse within 10 Gyr for *σ*/*m* = 50 cm^2^ g^−1^ even if it is isolated. In our *N*-body simulations, we consider three values, *σ*/*m* = 30 cm^2^ g^−1^ (SIDM30), 50 cm^2^ g^−1^ (SIDM50), and 100 cm^2^ g^−1^ (SIDM100), to explore a wide range of cross sections. A viable SIDM model should exhibit a velocity-dependent cross section that is large at low velocities while decreasing toward high velocities to evade constraints on massive halos around cluster scales ≲0.1 cm^2^ g^−1^ at ${V}_{{\mathrm{\max }}}\unicode{x0007E}1000\,{\mathrm{km}}\,{{\mathrm{s}}}^{-1}$ (A. H. G. Peter et al. 2013; M. Rocha et al. 2013; D. Harvey et al. 2015; M. Kaplinghat et al. 2016; K. E. Andrade et al. 2021; L. Sagunski et al. 2021; T. S. Ray et al. 2022; D. Kong et al. 2024). Nevertheless, for a specific halo, we can use a constant effective cross section to characterize its gravothermal evolution (D. Yang & H.-B. Yu 2022; N. J. Outmezguine et al. 2023; S. Yang et al. 2023b). In our case, ${V}_{{\mathrm{\max }}}$ decreases from ~15 to 7 km s^−1^ due to tidal mass loss. Thus, the *σ*/*m* values we consider can be regard as effective cross sections for ${V}_{{\mathrm{\max }}}\unicode{x0007E}10\,{\mathrm{km}}\,{{\mathrm{s}}}^{-1}$ on average, which overall align with SIDM models proposed to explain diverse dark matter distributions in galaxies (e.g., M. Valli & H.-B. Yu 2018; T. Ren et al. 2019; F. Kahlhoefer et al. 2019; M. Kaplinghat et al. 2019; J. Zavala et al. 2019; O. Sameie et al. 2020; C. A. Correa 2021; H. C. Turner et al. 2021; D. Yang & H.-B. Yu 2021; C. A. Correa et al. 2022; M. Silverman et al. 2022; D. Gilman et al. 2023; E. O. Nadler et al. 2023; O. Slone et al. 2023; M. S. Fischer et al. 2024b; M. Mancera Piña et al. 2024; A. Ragagnin et al. 2024; M. G. Roberts et al. 2024; X. Zhang et al. 2024; I. Dutra et al. 2025).

### 3.2. The Milky Way Model

The Milky Way is modeled as a static potential that contains three main components.

1. A spherical NFW halo:
  \begin{eqnarray*}{{\mathrm{\Phi }}}_{\,\mathrm{DM}\,}(r)=-4\pi G{\rho }_{s}{r}_{s}^{3}\frac{\mathrm{ln}\left(1+r/{r}_{s}\right)}{r},\end{eqnarray*}
  with *ρ*_*s*_ = 8.54 × 10^6^* M*_⊙_ kpc^−3^ and *r*_*s*_ = 19.6 kpc. *G* is the Newton constant.
2. A spherical stellar bulge with a Hernquist profile (L. Hernquist 1990):
  \begin{eqnarray*}{{\mathrm{\Phi }}}_{{\mathrm{b}}}(r)=-\frac{G{M}_{{\mathrm{b}}}}{{r}_{{\mathrm{H}}}+r},\end{eqnarray*}
  with *M*_b_ = 9.23 × 10^9^* M*_⊙_ and *r*_H_ = 1.3 kpc.
3. Two stellar disks and two gas disks with an axisymmetric Miyamoto–Nagai profile (M. Miyamoto & R. Nagai 1975):
  \begin{eqnarray*}{{\mathrm{\Phi }}}_{{\mathrm{}}}(R,z)=-\frac{G{M}_{{\mathrm{d}}}}{{\left[{R}^{2}+{\left({a}_{{\mathrm{d}}}+\sqrt{{z}^{2}+{b}_{{\mathrm{d}}}^{2}}\right)}^{2}\right]}^{1/2}}.\end{eqnarray*}
  The parameters for each disk are as follows. Thin stellar disk: *M*_d_ = 3.52 × 10^10^* M*_⊙_, *a*_d_ = 2.50 kpc, and *b*_d_ = 0.3 kpc; thick stellar disk: *M*_d_ = 1.05 × 10^10^* M*_⊙_, *a*_d_ = 3.02 kpc, and *b*_d_ = 0.9 kpc; thin gas disk: *M*_d_ =1.2 × 10^9^* M*_⊙_, *a*_d_ = 1.5 kpc, and *b*_d_ = 0.045 kpc; and thick gas disk: *M*_d_ = 1.1 × 10^10^* M*_⊙_, *a*_d_ = 7.0 kpc, and *b*_d_ = 0.085 kpc.

These parameters are motivated by the Milky Way mass model in P. J. McMillan (2016). Note that the stellar and disk density profiles in P. J. McMillan (2016) use exponential functions, which are challenging to implement in controlled *N*-body simulations due to the lack of analytical expressions for their corresponding potentials. Nevertheless, we have verified that the difference in the total potential remains within 2% in the regions with $\sqrt{{R}^{2}+{z}^{2}}> 10\,{\mathrm{kpc}}$, which are most relevant for our simulated subhalo. Since the host halo is treated as a static potential, we neglect dark matter particle scatterings between the host halo and the subhalo. This approximation is well justified for velocity-dependent SIDM models with *σ*/*m* ≲ 1 cm^2^ g^−1^ at ${V}_{{\mathrm{\max }}}\unicode{x0007E}200\,{\mathrm{km}}\,{{\mathrm{s}}}^{-1}$ (E. O. Nadler et al. 2020a).

### 3.3. Orbital Parameters

A. Bonaca et al. (2020) found that the best-fit orbit of GD-1 has a pericenter of *r*_peri_ = 13.8 kpc and an apocenter of *r*_apo_ = 22.3 kpc, while the orbit of its perturber remains highly uncertain. For our simulation, we adopt an orbit with *r*_peri_ = 17 kpc and *r*_apo_ = 142 kpc, with the simulated subhalo undergoing five pericenter passages over 10 Gyr. Although we do not aim to explicitly model the encounter event, at *t* ≈ 10 Gyr, the simulated subhalo's coordinates are R.A. = 21$\mathop{.}\limits^{\unicode{x000b0}}$5 and decl. = −7$\mathop{.}\limits^{\unicode{x000b0}}$9, consistent with the inferred position range of the present-day GD-1 perturber (A. Bonaca et al. 2020). While this orbit differs from that of the progenitor halo selected from the cosmological merger tree (D. Yang et al. 2023a), it remains typical for many subhalos in the simulation. We emphasize that gravothermal collapse is intrinsic to SIDM halos, and the overall properties of our simulated perturber are robust regardless of the specific orbit chosen.

## 4. Results

**Figure 2.**
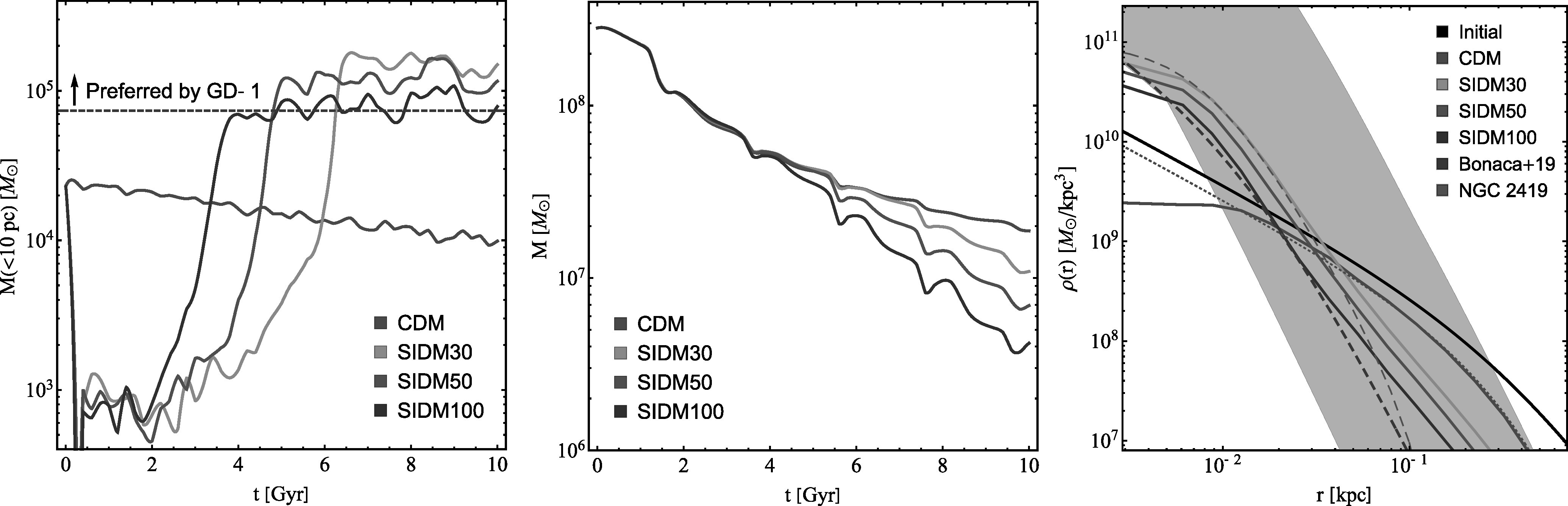
Left: evolution of the enclosed mass within 10 pc for the CDM (blue), SIDM30 (amber), SIDM50 (orange), and SIDM100 (pink) subhalos. The horizontal dashed gray line denotes the inner mass within 10 pc of the reference Hernquist profile, as shown in Figure 1 (middle). Middle: evolution of the bound mass for the simulated CDM and SIDM subhalos. Right: corresponding density profiles for the simulated CDM and SIDM subhalos at *t* = 10 Gyr, along with the initial NFW profile (black). The dotted blue line denotes a reconstructed density profile for the CDM subhalo using an analytical function proposed by R. Errani & J. F. Navarro (2021). The gray shaded region denotes the viable range for the GD-1 perturber, converted from Figure 6 of A. Bonaca et al. (2019), while the dashed gray line represents the reference Hernquist profile. The dashed cyan line represents the density profile of the globular cluster NGC 2419, modeled using the King profile from H. Baumgardt et al. (2009).

Figure 2 (left) shows the evolution of the enclosed mass within the inner *r* = 10 pc for CDM (blue), SIDM30 (amber), SIDM50 (orange), and SIDM100 (pink) subhalos. For CDM, the inner mass decreases monotonically due to tidal stripping. In contrast, for SIDM, the mass initially decreases sharply due to core expansion, followed by an increase as core collapse occurs. By *t* ≈ 10 Gyr, the inner mass of the SIDM subhalos is 1 order of magnitude higher than that of the CDM subhalo, aligning well with the reference Hernquist profile (horizontal line). Additionally, the collapse times are *t*_*c*_ ~ 6, 4, and 2 Gyr for SIDM30, SIDM50, and SIDM100, respectively, about a factor of 2 shorter than those estimated using Equation 2, which is calibrated for isolated halos. In a subhalo, tidal stripping reduces the velocity dispersion of dark matter particles from the intermediate to outer regions as a result of mass loss. Consequently, a negative “temperature” gradient—a necessary condition for the onset of core collapse—is more easily established compared to an isolated halo (O. Sameie et al. 2020).

In Figure 2 (middle), we show the evolution of the total bound mass for the simulated CDM and SIDM subhalos. Initially, the halo mass is 3.25 × 10^8^* M*_⊙_ and is reduced by 1 order of magnitude by *t* ≈ 10 Gyr due to tidal stripping. As expected, the total mass loss is more significant as the cross section increases. For SIDM, the final halo mass ranges from 4 × 10^6^ to 10^7^* M*_⊙_, which falls well within the favored mass range of the GD-1 perturber 3 × 10^5^–10^8^ *M*_⊙_ (A. Bonaca et al. 2019).

Figure 2 (right) shows the corresponding density profiles at *t* = 10 Gyr for the CDM and SIDM subhalos, along with the initial NFW profile. For comparison, the viable region for the GD-1 perturber (shaded gray), converted from Figure 6 of A. Bonaca et al. (2019), and the reference Hernquist profile (dashed gray) are also shown. Compared to CDM, the density profiles of the SIDM subhalos are significantly steeper and overall consistent with the favored Hernquist profiles from A. Bonaca et al. (2019). This indicates that dark matter self-interactions can both increase central density and accelerate tidal mass loss in the outer regions. Consequently, an SIDM subhalo can become more compact and dense than its CDM counterpart.

We note that the CDM subhalo has a small density core near the center. This is due to the resolution limit as *ℓ* = 2.8*ϵ* ≈ 5.6 pc, although the simulated subhalo contains more than 6 × 10^5^ simulation particles at *t* = 10 Gyr. We fit the density profile using the analytical function $\rho (r)={\rho }_{{\mathrm{cut}}}\exp (-r/{r}_{{\mathrm{cut}}}){r}_{{\mathrm{cut}}}/r$ from R. Errani & J. F. Navarro (2021), which is proposed to model a tidally stripped CDM halo. With *ρ*_cut_ ≈ 1.2 × 10^8^* M*_⊙_ kpc^−3^ and *r*_cut_ ≈ 0.22 kpc, we find a good fit for the region *r* ≳ 10 pc; see Figure 2 (right, dotted blue). The fitted function has a cusp *ρ*(*r*) ∝ *r*^−1^ near the center, and it provides a correction to the core due to the resolution limit. For the fitted profile, the enclosed mass within 10 pc is 1.6 × 10^4^* M*_⊙_. However, even with this correction, the CDM subhalo remains insufficiently dense to explain the high density of the GD-1 perturber.

In Figure 2 (right), we also present the density profile of the globular cluster NGC 2419 (cyan), modeled using the King profile from H. Baumgardt et al. (2009). Interestingly, this profile closely resembles the density profile of the SIDM30 subhalo within 30 pc. This similarity is not coincidental, as the formation of globular clusters follows the same mechanism as the collapse of SIDM halos. This suggests that distinguishing between SIDM and globular cluster scenarios in explaining the GD-1 perturbation could be challenging. However, NGC 2419 itself cannot be the GD-1 perturber, as its orbit does not align with the perturbation (A. Bonaca et al. 2019). If the perturbation is caused by an undetected globular cluster that emits light, it could be identified in future astronomical surveys.

Furthermore, narrowing down the favored parameter space in the mass–size plane for the perturber would help us distinguish the two scenarios. For instance, if the perturber's mass is further constrained to the range 10^7^–10^8^* M*_⊙_, the SIDM scenario would be favored, as globular clusters typically have masses below a few times 10^6^* M*_⊙_. Another intriguing possibility is that the perturber is an SIDM substructure hosting stars, as we will discuss later. It may have undergone significant tidal stripping, resulting in an ultrafaint dwarf with mass and structural properties similar to those of a massive globular cluster (S. Mau et al. 2020). Confirming this scenario would require detecting a stellar counterpart at the inferred location of the perturber. Distinguishing between these possibilities will require dedicated observational campaigns and detailed modeling efforts, making this an exciting avenue for future research.

## 5. Discussions and Conclusion

The inner density profiles of our simulated SIDM subhalos (*r* ≲ 10 pc) could be underestimated due to numerical issues in *N*-body simulations when the halo is deeply collapsed (Y.-M. Zhong et al. 2023; M. S. Fischer et al. 2024a; C. Mace et al. 2024; I. Palubski et al. 2024). Specially, numerical artifacts introduce additional “energy” that heats the simulated halo, slowing down or even preventing further increases in inner density; see M. S. Fischer et al. (2024a) for discussions about potential causes. As shown in Figure 2 (left), for the SIDM subhalos, the enclosed mass within inner 10 pc stalls after *t* ≈ 4.5–6.5 Gyr, suggesting that they may suffer from the artificial heating effect. To further test this, we conducted an isolated simulation without the tidal field for the same initial NFW profile and *σ*/*m* = 50 cm^2^ g^−1^. Since the isolated halo experiences no tidal mass loss or heating, its total energy can be computed straightforwardly. See Appendix A for details on the isolated simulation and comparison with the subhalos.

Indeed, we find that the energy increases when the isolated halo enters the deep collapse phase, corresponding to a Knudsen number of *Kn* ≈ 0.4 within 10 pc, i.e., the ratio of the mean free path to the gravitational height (e.g., S. Balberg et al. 2002; R. Essig et al. 2019). For the SIDM subhalos, the stalling behavior occurs when their *Kn* values reach 0.3–0.6. In comparison, the total energy of the simulated SIDM halo in M. S. Fischer et al. (2024a; their Figure 1) starts to increase when *Kn* reaches 0.1. Even at *Kn* = 0.01 energy conservation violation is at the 1.5% level, better than our simulation. This is likely because M. S. Fischer et al. (2024a) adopted a more accurate criterion for the gravity computations while at a higher computational cost. Since the artificial heating effect leads to an underestimation of the inner density profile for a collapsed SIDM halo, our results are conservative in this regard. Nevertheless, it will be important to further improve the SIDM prediction as future measurements of the GD-1 stream could narrow down the viable parameter space of the perturber (A. Bonaca & A. M. Price-Whelan 2024).

When modeling the Milky Way, we used static potentials for both halo and stars, calibrated with present-day measurements. Simulations show that Milky Way–like systems could grow significantly over the last ~6 Gyr due to mergers and accretion (e.g., M. Ishchenko et al. 2023; Y. Wang et al. 2024). If these effects were incorporated, our simulated subhalo would experience weaker tidal stripping in the early stages. However, we note that this is degenerate with the orbital parameters; similar results can be achieved by lowering the pericenter if a weaker potential is adopted at early times. In Appendix A, we will see that even for an isolated halo, the SIDM50 case can still collapse to the viable parameter region. Additionally, encounters between the GD-1 stream and the perturber are likely to have occurred within the last 1 Gyr (A. Bonaca et al. 2019), and hence the growth history of the Milky Way may not directly impact the inference of the perturber's properties.

The subhalo we used to demonstrate the SIDM scenario for the GD-1 perturber has an infall mass of ≈3 × 10^8^* M*_⊙_. Interestingly, this is near the upper limit on the peak mass of subhalos that host currently observed satellite galaxies in the Milky Way (P. Jethwa et al. 2018; E. O. Nadler et al. 2020b). Thus, it remains an open question whether the perturber is a truly dark substructure, devoid of a galaxy. To further investigate detectability, we conducted additional simulations for the CDM and SIDM50 cases with live stellar particles, assuming a Plummer stellar profile with a scale radius of 0.3 kpc and a total mass of 3.2 × 10^4^* M*_⊙_, motivated by hydrodynamical simulations of the Local Group (A. Fattahi et al. 2018). At *t* = 10 Gyr, the bound stellar masses are 1.8 × 10^4^ and 1.3 × 10^4^* M*_⊙_ for the CDM and SIDM50 cases, respectively, with the latter also exhibiting a steeper stellar density profile toward the central regions. These substructures fall into the category of ultrafaint dwarf galaxies and could potentially be detected in the near future through observations, e.g., with the Rubin Observatory (A. Drlica-Wagner et al. 2019; Ž. Ivezić et al. 2019).

More work is needed along these lines. For instance, the stellar–halo mass relation becomes increasingly steep in the ultrafaint regime and exhibits significant scatter (A. Fattahi et al. 2018), which must be taken into account. Additionally, since the Rubin Observatory can only detect objects in the southern hemisphere, it would be crucial to assess Rubin's sky coverage in conjunction with the orbital information of the GD-1 perturber from A. Bonaca et al. (2020). We leave these investigations for future work. Furthermore, our scenario should also apply to smaller infall masses below ~10^8^* M*_⊙_. Indeed, for SIDM models with large velocity-dependent cross sections, the population of core-collapsing (sub)halos increases as the mass decreases (e.g., E. O. Nadler et al. 2023; D. Yang et al. 2023a). Thus, stellar streams like GD-1 can probe both population and density profile of core-collapsing subhalos even below the mass threshold for galaxy formation.

We used the Hernquist profile for the GD-1 perturber from A. Bonaca et al. (2019) as a reference to assess the simulated subhalos. It would be intriguing to take the SIDM subhalo and directly model its encounter with GD-1, incorporating the influence of the Large Magellanic Cloud (e.g., D. Erkal et al. 2019; N. Shipp et al. 2021). We could use the parametric model (S. Ando et al. 2024; D. Yang et al. 2024a, 2024b) to generate a population of collapsed SIDM subhalos in Milky Way analogs. To overcome the numerical issues in *N*-body simulations of core-collapsing halos, we may complement them with the semianalytical fluid model to better capture the dynamics in the the central regions (e.g., S. Balberg et al. 2002; Y.-M. Zhong et al. 2023; S. Gad-Nasr et al. 2024).

In summary, we have conducted controlled *N*-body simulations and shown that a core-collapsed SIDM halo could explain the high density of the GD-1 stellar stream perturber. For progenitor halos from the cosmological simulation of a Milky Way analog, the required self-interacting cross section *σ*/*m* ≳ 30 cm^2^ g^−1^ for ~10^8^* M*_⊙_ halos with ${V}_{{\mathrm{\max }}}\unicode{x0007E}10\,{\mathrm{km}}\,{{\mathrm{s}}}^{-1}$. Dark matter self-interactions can both increase inner density and accelerate tidal mass loss in the outer regions, producing a compact and dense perturber to explain the spur and gap features of the GD-1 stream. Our findings demonstrate that stellar streams provide a novel probe into the self-interacting nature of dark matter. We have also outlined future investigations to further improve this promising approach.

## Figures and Tables

**Figure 3.**
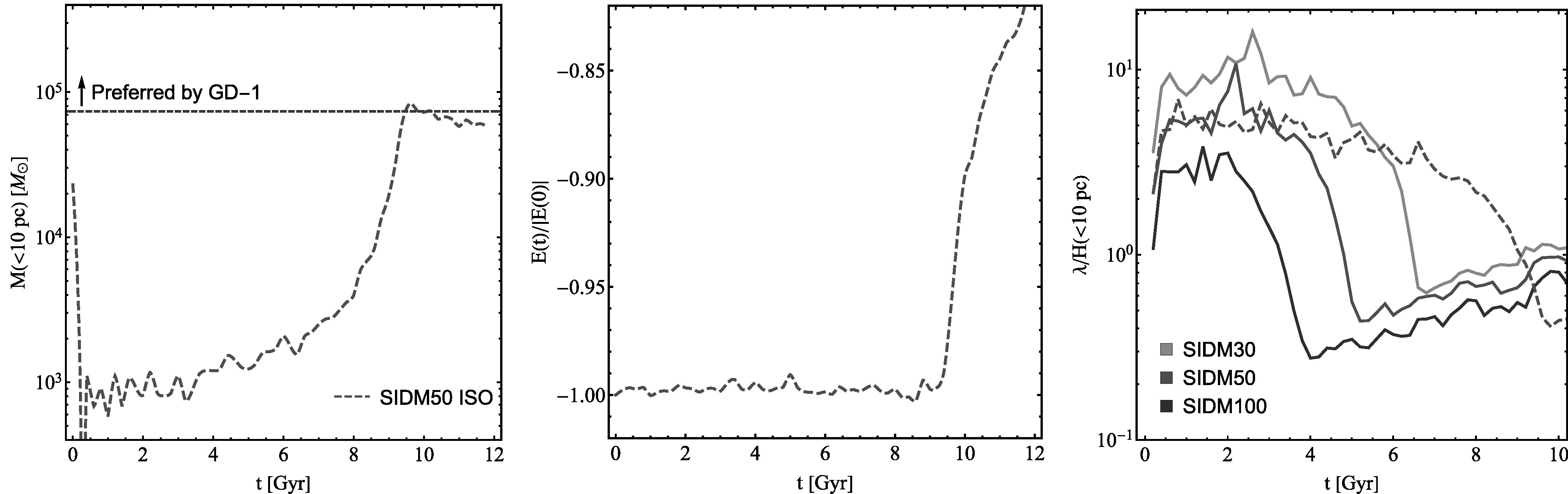
Left: evolution of the enclosed mass within 10 pc for the SIDM50 isolated halo without evolving in the tidal field (dashed orange). The horizontal line denotes the Hernquist profile as in Figure 2 (left). Middle: evolution of the total energy normalized to its initial absolute value for the SIDM50 isolated halo. Right: evolution of the ratio *λ*/*H* within inner 10 pc for the SIDM50 isolated halo (dashed orange), along with the SIDM30 (solid amber), SIDM50 (solid orange), and SIDM100 (solid pink) subhalos.

**Figure 4.**
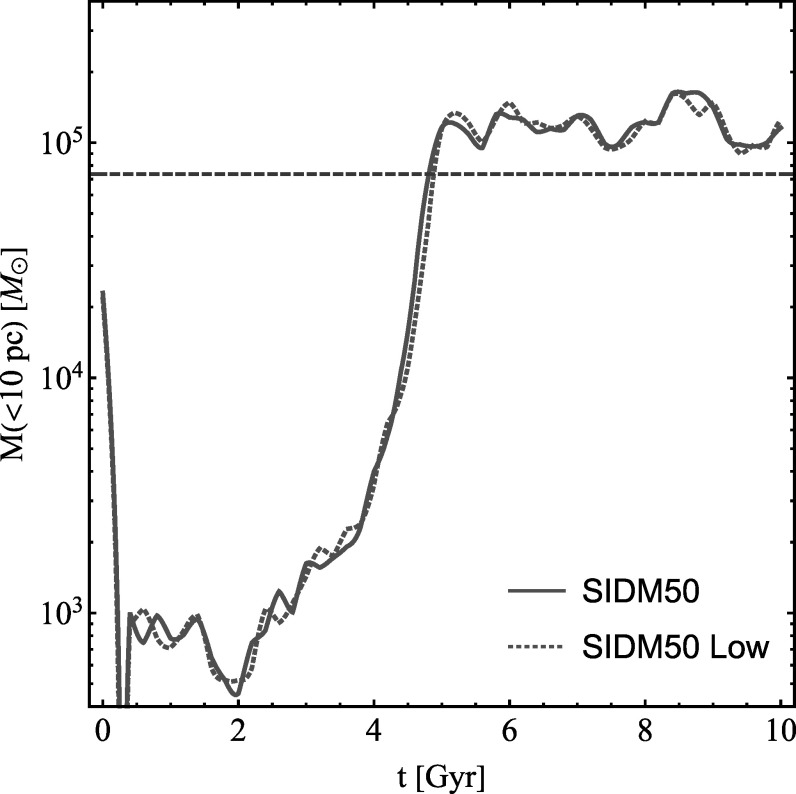
The evolution of the enclosed mass within inner *r* = 10 pc for the low-resolution (dotted orange) and high-resolution (solid orange) simulations.

## Appendix A Simulating Halos in the Deep Collapse Phase

*N*-body simulations of a core-collapsing SIDM halo are challenging (Y.-M. Zhong et al. 2023; M. S. Fischer et al. 2024a; C. Mace et al. 2024; I. Palubski et al. 2024). In particular, when a halo enters the phase of deep collapse, energy conservation can be violated in the simulation, resulting in an increase in total energy due to numerical artifacts; see M. S. Fischer et al. (2024a) for discussions on potential causes. This “heating” effect slows down the further collapse of the simulated halo and the increase of its inner density. As shown in Figure 2 (left), the enclosed mass of the SIDM subhalos stalls at late stages, indicating that they may be affected by these numerical artifacts. To assess the condition of energy conservation, we simulate an *isolated* halo with the same initial NFW profile and *σ*/*m* = 50 cm^2^ g^−1^, without evolving it in the tidal field. For an isolated halo, it is straightforward to evaluate its total energy over time, allowing us to test our simulation setup.

In Figure 3 (left), we illustrate the evolution of the enclosed mass within the inner 10 pc of the isolated SIDM50 halo. The overall behavior is similar to that of the SIDM50 subhalo presented in Figure 2 (left), but the collapse timescale for the isolated halo is approximately a factor of 2 longer due to the absence of tidal acceleration. After the inner mass reaches its peak at *t* ≈ 9.5 Gyr, it stops increasing and instead experiences a slight decrease. Figure 3 (middle) shows the evolution of the total energy of the isolated halo, normalized to its initial absolute value. We observe that the total energy deviates significantly from its initial value for *t* > 9.5 Gyr due to the artificial heating effect.

To further clarify this issue, we compute the Knudsen number *Kn* ≡ *λ*/*H*, where *λ* is the mean free path and *H* is the gravitational scale height, i.e.,

\begin{eqnarray*}\lambda =\displaystyle \frac{1}{\rho \sigma /m},\,H=\sqrt{\displaystyle \frac{{\sigma }_{{\mathrm{v}}}^{2}}{4\pi G\rho }},\end{eqnarray*}

where *G* is Newton's constant and *σ*_v_ is the 1D velocity dispersion of dark matter particles. Figure 3 (right) shows the evolution of the Knudsen number averaged over inner 10 pc for the SIDM50 isolated halo (dashed orange), and the SIDM30 (solid amber), SIDM50 (solid orange), and SIDM100 (solid pink) subhalos. At *t* ≈ 9.5 Gyr, the corresponding Knudsen number is *Kn* ≈ 0.4 for the isolated halo. Thus, we expect that the “heating” effect becomes an issue for our simulation when *Kn* is close to 0.4. For the SIDM subhalos, their lowest *Kn* value ranges from 0.3 to 0.6. This may explain why their enclosed mass stalls after *t* ≈ 4.5–6.5 Gyr.

It is useful to compare to the halo presented in M. S. Fischer et al. (2024a), where they simulated a 1.2 × 10^11^ *M*_⊙_ isolated halo assuming *σ*/*m* = 100 cm^2^ g^−1^. The particle mass is 3 × 10^4^ and the softening length is *ϵ* = 0.13 kpc. In that simulation, the energy starts to increase at *t* ≈ 9.6 Gyr (their Figure 1), corresponding to *Kn* ≈ 0.1. However, even at *Kn* = 0.01 energy conservation violation is at only the 1.5% level, better than our simulation. This difference is likely because M. S. Fischer et al. (2024a) used a more accurate cell-opening criterion for the gravity computations (ErrTolForceAcc = 5 × 10^−4^) compared to ours (ErrTolForceAcc = 5 × 10^−3^), with the former being more computationally expensive. Since the artificial heating effect leads to an underestimation of the inner density profile for a collapsed SIDM halo, our results are conservative.

## Appendix B Convergence

Lastly, to check the convergence, we have conducted an additional simulation for the SIDM50 subhalo with the total number of particles *N* = 5 × 10^6^, a factor of 2 smaller than that used to produce our main results. Figure 4 shows the evolution of the enclosed mass within an inner radius *r* = 10 pc with the low-resolution (dotted orange) and high-resolution (solid orange) simulations. The results converge well. As discussed in Appendix A, the stalling behavior of the enclosed mass indicates that the energy conservation is violated due to the artificial heating effect. We see both high- and low-resolution simulations suffer from this issue, despite their convergence.
